# Acetaminophen Disrupts the Development of Pharyngeal Arch-Derived Cartilage and Muscle in Zebrafish

**DOI:** 10.3390/jdb10030030

**Published:** 2022-07-14

**Authors:** Derrick M. Glasco, Zhidong Wang, Seonwoo Kang, Avery T. Funkhouser

**Affiliations:** 1 Department of Biology, Division of Natural Sciences, Bob Jones University, Greenville, SC 29614, USA; zhiwang@augusta.edu (Z.W.); skang598@postech.ac.kr (S.K.); averytf@email.sc.edu (A.T.F.); 2 Department of Chemistry, Postech-Catholic Biomedical Engineering Institute, School of Interdisciplinary Bioscience and Bioengineering, Pohang University of Science and Technology, Pohang 37673, Korea; 3 School of Medicine Greenville, University of South Carolina, Greenville, SC 29605, USA

**Keywords:** zebrafish, craniofacial development, acetaminophen, chondrogenesis, musculogenesis, neural crest, pharyngeal arches

## Abstract

Acetaminophen is a common analgesic, but its potential effects on early embryonic development are not well understood. Previous studies using zebrafish *(Danio rerio)* have described the effects of acetaminophen on liver development and physiology, and a few have described gross physiological and morphological defects. Using a high but non-embryonic lethal dose of acetaminophen, we probed for defects in zebrafish craniofacial cartilage development. Strikingly, acetaminophen treatment caused severe craniofacial cartilage defects, primarily affecting both the presence and morphology of pharyngeal arch-derived cartilages of the viscerocranium. Delaying acetaminophen treatment restored developing cartilages in an order correlated with their corresponding pharyngeal arches, suggesting that acetaminophen may target pharyngeal arch development. Craniofacial cartilages are derived from cranial neural crest cells; however, many neural crest cells were still seen along their expected migration paths, and most remaining cartilage precursors expressed the neural crest markers sox9a and sox10, then eventually col2a1 (type II collagen). Therefore, the defects are not primarily due to an early breakdown of neural crest or cartilage differentiation. Instead, apoptosis is increased around the developing pharyngeal arches prior to chondrogenesis, further suggesting that acetaminophen may target pharyngeal arch development. Many craniofacial muscles, which develop in close proximity to the affected cartilages, were also absent in treated larvae. Taken together, these results suggest that high amounts of acetaminophen can disrupt multiple aspects of craniofacial development in zebrafish.

## 1. Introduction

The zebrafish is a well-established model organism in developmental toxicology and has similar success as mammalian models in assessing developmental toxicity [1]. They readily absorb compounds from the environment, and their rapid, translucent development make them ideally suited for probing for developmental defects at the cellular through organismal levels. Furthermore, since genetic and developmental mechanisms are similar between zebrafish and mammals [2,3], they often serve as a valuable starting point for analysis. In this study, we examined the effects of acetaminophen (APAP) on embryonic development using zebrafish. The hepatotoxic effects of APAP overdose are well known [4], but knowledge of its potential effects on embryonic development is limited. Importantly, zebrafish possess many drug-metabolizing enzymes [5], including those homologous to APAP-metabolizing enzymes in other vertebrates [6]. In the zebrafish liver, APAP undergoes CYP-mediated conversion to N-acetyl-p-benzoquinone imine (NAPQI) [7]. NAPQI is typically benign in low amounts where it can be efficiently excreted but has hepatotoxic properties upon APAP overdose [4].

Previous studies using zebrafish have reported gross morphological and physiological defects as a result of APAP exposure, including spinal curvature, reduced body size and pigmentation, deformed tail and swim bladder, pericardial and peritoneal edema, delayed hatching, and abnormal heart rate [8,9,10,11,12,13,14,15,16]. Their obvious reduction in head size was particularly intriguing to us and led us to examine craniofacial development in APAP-treated larvae.

Craniofacial development involves a complex set of processes in which errors can lead to congenital defects. The skull consists of the neurocranium, comprising the bones protecting the brain and brain stem, and the viscerocranium, comprising the bones of the face and jaw. Defects in viscerocranial development can result in orofacial clefts [17]. In vertebrates, the craniofacial skeleton is derived primarily from Sox9-expressing cranial neural crest cells (NCCs), many of which condense and differentiate into chondrocytes and secrete type II collagen (Col2a1 gene). As development progresses, most craniofacial cartilages are replaced by bone through endochondral ossification, while other bones are directly derived from stem cell precursors through intramembranous ossification. In zebrafish, viscerocranial cartilages are derived from NCCs that populate the pharyngeal arches [18,19]. The first arch gives rise to Meckel’s cartilage, the second arch gives rise to the ventral ceratohyal and dorsal hyosymplectic cartilages, and the third through seventh arches give rise to the ceratobranchial cartilages. Neurocranial cartilages, notably the ethmoid plate and trabecular cartilages, are derived from NCCs that migrate from the first pharyngeal arch and into the neurocranium [18,20].

Using zebrafish as a model, we report here that in addition to numerous morphological and physiological defects, overexposure to APAP causes severe craniofacial cartilage defects. Intriguingly, cartilages derived from the viscerocranium were primarily affected, but not those of the neurocranium. While many cartilage types are missing in APAP-treated larvae, the remaining cartilages properly express NCC markers, arguing against NCC differentiation as an underlying cause. Instead, patterns of apoptosis in early embryos suggest that APAP may hinder pharyngeal arch development, upstream of chondrogenesis. These findings, combined with our attempts to recapitulate the defects in the mouse model, give insight into how APAP can affect craniofacial development.

## 2. Materials and Methods

### 2.1. Zebrafish Embryo Collection and Treatment

Zebrafish *(Danio rerio)* were maintained in accordance with The Zebrafish Book [21], and all methods were approved by the Institutional Animal Care and Use Committee at Bob Jones University. Adults were housed in 10-gallon tanks on a 14/10 light/dark cycle at 28.5 °C and a pH of 6.5–7. Health and behavior were monitored twice daily, and individuals with any sign of disease or abnormal behavior were euthanized immediately using the hypothermal shock method [22].

For embryo collection, tanks were marbled overnight, and fertilized eggs were siphoned immediately after laying. Embryos were grown at 28.5 °C in E3 embryo medium. Treated embryos were grown in 3.9-mM APAP (4-acetamidophenol, Alfa Aesar, A1124030, Ward Hill, MA, USA) or a non-hepatotoxic analog (3-acetamidophenol, Alfa Aesar, A1276414, Ward Hill, MA, USA), dissolved in E3 medium from 2 hpf continually, unless otherwise indicated. Media were replaced daily for all control and treatment groups.

To block pigmentation of embryos for cartilage staining, immunohistochemistry, acridine orange staining, or GFP transgene visualization (each described below), E3 media was supplemented with 0.2-mM PTU starting at 24 hpf. For all other assays, embryos were allowed to pigment normally.

*Tg(mylz2:GFP)* “GloFish” [23,24] were obtained from a local vendor, housed separately from wild-type fish, and used exclusively for the visualization of developing muscles.

### 2.2. Cartilage Staining

To visualize cartilage, 6-dpf larvae were stained with alcian blue as previously described [25]. Larvae were fixed in 4% PFA overnight, washed with 0.1% Tween-20 in PBS, bleached with 30% hydrogen peroxide for 2 h, washed again, then incubated in alcian blue (0.1% alcian blue, 1% HCl, 70% ethanol in PBS) overnight. They were then washed four times in acidic ethanol (5% HCl, 70% ethanol in PBS) for 1 h each and stored in 70% glycerol for imaging.

### 2.3. Whole-Mount Immunohistochemistry

Embryos were fixed in 4:1 methanol:DMSO overnight at 4 °C, then stored in 100% methanol at −20 °C for up to several weeks. To begin, samples were rehydrated by washing in 66% methanol in PBS, 33% methanol in PBS, then PBS for 30 min each. (Exception: embryos processed for caspase-3 staining were fixed in 4% PFA at 4 °C overnight, then washed only in PBS). Nonspecific antibody binding was blocked with two washes of PBSMT (2% Carnation™ skim milk powder, 0.1% Triton X-100 in PBS) for 1 h each at room temperature, followed by incubation with the primary antibody diluted in PBSMT overnight at 4 °C. The next day, they were washed with PBSMT twice for 1 h each at 4 °C, followed by three room temperature washes for 1 h each. Samples were then incubated with the secondary antibody diluted in PBSMT overnight at 4 °C in the dark. Samples were again washed with PBSMT twice for 1 h each at 4 °C, followed by three room temperature washes for 1 h each. To prepare for imaging, samples were washed twice with PBS for 10 min each, then mounted in glycerol for imaging.

The following primary antibodies were used: Monoclonal rabbit anti-activated Caspase-3, 1:100 (BD 559565); monoclonal mouse anti-COL2A1, 1:200 (Developmental Studies Hybridoma Bank [DSHB], II-II6B3, deposited by Linsenmayer, T.F.); polyclonal rabbit anti-Sox9a N-terminal, 1:200 (Abcam, ab209820); monoclonal mouse anti-SOX10, 1:50 (DSHB, PCR-SOX10-1D8, deposited by the Protein Capture Reagents Program, produced by JHU/CDI); and monoclonal mouse anti-Titin, 1:50 (DSHB, 9 D10, deposited by Greaser, M.L.). The following secondary antibodies were used: Polyclonal goat anti-mouse Alexa Fluor 488 at 1:200 (Invitrogen, A-11001, Waltham, MA, USA) and polyclonal goat anti-rabbit Alexa Fluor 488 at 1:200 (Invitrogen, A-11034, Waltham, MA, USA).

### 2.4. Live Imaging of Embryos and Larvae

To visualize apoptotic cells in live embryos, acridine orange staining was performed as previously described [26]. Dechorionated 24-hpf embryos were incubated in 5 µg/mL acridine orange (Sigma-Aldrich, A6014, St. Louis, MO, USA) in E3 for 30 min at room temperature, then washed several times with E3 to eliminate excess stain. For imaging, individual embryos were moved to 0.6-mM tricaine (Sigma-Aldrich, A5040, St. Louis, MO, USA) in E3 to reduce movement, then imaged using a compound epi-fluorescence microscope. After imaging, the embryos were immediately euthanized in 4% PFA.

To visualize developing muscles in *mylz2:GFP* fish, larvae were briefly anesthetized with 0.6-mM tricaine in E3, and imaged using an epi-fluorescence stereo microscope. 

### 2.5. Analysis of Fluorescence Intensity in Anti-Activated Caspase-3-Labeled Embryos 

To quantify the differences in levels of apoptosis between untreated and APAP-treated embryos, images were analyzed using ImageJ software (https://imagej.nih.gov/ij/, accessed on 13 October 2021). Using the polygon selections tool in ImageJ, specific anatomical regions for each type of view were outlined for analysis. For lateral views of the head, the entire head through the posterior end of the otic vesicle was selected. For ventral views of the head, the entire head anterior to the yolk was selected. For lateral views of the trunk, the region directly above the entire yolk extension was selected. To measure pixel intensity within the outlined regions, the mean gray value was calculated (ImageJ: Analyze, then Measure). To ensure accuracy in comparing pixel intensity (arbitrary units ranging from 0–255), raw images between groups to be compared were captured using identical camera settings, and all images were pre-checked to verify lack of saturation (ImageJ: Analyze, then Histogram = max pixel intensity of <255). To subtract background signal, the mean pixel intensity of identical negative control images (embryos processed in parallel without a primary antibody) were subtracted from the original values. The resulting values reflect the increase in fluorescence above the negative controls.

### 2.6. Touch Response Assay

The touch response assay was performed as previously described [27]. Each 2-dpf embryo was placed into a glass dish and aligned to the center of a 20-mm field of view under a stereo microscope. Using a fine insect pin, a single touch was applied to the head or trunk of each embryo. Responses were captured on video to ensure accurate analysis and sorted into one of three categories: No Response (no movement upon stimulus), Weak Response (movement away from the stimulus but remaining within the field of view), and Strong Response (movement away from the stimulus and leaving the field of view). Chi-square tests were performed to detect any significant differences between untreated and APAP-treated groups for head and trunk touches.

### 2.7. Analysis of Survivability, Heart Rate, and Hatching Behavior

To track survivability and heart rates, APAP-treated (3.9-mM APAP in E3) or untreated (E3 only) embryos were grown in groups of up to 12 in glass petri dishes. Every 12 h (8 AM and 8 PM), all embryos were visually inspected, and heart rates were calculated by recording the number of beats in 10 s and multiplying by six. Embryos were dechorionated when necessary to assess heart rate. Any dead embryos, as defined by a lack of heartbeat, were removed. Media were replaced daily. Beginning at 5 dpf, dishes were supplemented with an appropriate larval food to ensure that any death was not caused by lack of a food source.

To track hatching behavior in separate experiments, embryos were treated as above except they were not dechorionated. Embryos were considered to have hatched when they had completely broken free from the chorion.

### 2.8. Mouse APAP Administration by Oral Gavage

Mice *(Mus musculus)* were obtained from a local vendor and maintained in accordance with the Guide for the Care and Use of Laboratory Animals, 8th edition (2011). All methods were approved by the Institutional Animal Care and Use Committee at Bob Jones University, and all effort was made to minimize pain and suffering. Males were housed individually and females were housed in groups of four in 30 × 19 × 19-cm mouse cages on a 14:10 light:dark cycle in a dedicated facility. Welfare checks were performed daily, and any mice showing injury or distressed behavior (*n* = 2 in early trials) were euthanized immediately via exposure to 5% isoflurane for 10 min, followed by cervical dislocation. The total number of adult mice used in this study was approximately 40, housed over a period of five months. Upon completion, mice were used as colony breeders. D.M.G. performed the mouse experiments and received training from workshops at the University of Missouri-Columbia. 

Wild-type female mice were timed-mated with wild-type studs, and their weight was monitored daily as an indicator of health and to verify pregnancy when possible. Starting at the appropriate timepoint (either E7.25 or E10.25, with E0.5 being noon on the day of observing a copulation plug), pregnant females were administered either 0.9% saline or 200 mg/kg APAP three times daily (approximately 8 AM, 2 PM, and 8 PM) for four days via oral gavage. APAP was administered in the form of Infants’ Tylenol at 160 mg/5 mL [28]. Females were briefly anesthetized with 2% isoflurane prior to each dose [29], then gavaged using a 20-gauge × 33-mm flexible gavage needle (gavageneedle.com, 2038NB). They were then monitored for 15 min after each dose to ensure a return to normal activity. At the end of four days of APAP or saline administration, females were allowed to continue pregnancy as normal. The resulting pups were euthanized at postnatal day 0 (P0) by exposure to 5% isoflurane for 50 min, then immediately frozen until use in skeletal staining.

For comparative purposes, the human equivalent dose was calculated using FDA recommendations (https://www.fda.gov/media/72309/download, accessed on 1 September 2020) assuming a 60-kg adult.

### 2.9. Mouse Pup Skeletal Staining

Whole mount skeletal staining was performed as previously described [30]. Euthanized P0 pups were dissected to remove all skin, internal organs, and fatty tissue. All subsequent steps were performed on an orbital rocker at room temperature. The preps were fixed overnight in 95% EtOH, then moved to 100% acetone overnight. To stain cartilage, pups were incubated in alcian blue solution (0.03% alcian blue 8GX [Alfa Aesar, J60122, Ward Hill, MA, USA], 80% EtOH, 20% glacial acetic acid) overnight. They were then washed twice with 70% EtOH in PBS over the course of a day, then overnight in 95% EtOH. Pups were then cleared with 1% KOH in PBS for 1 h. To stain bone, pups were incubated in alizarin red solution (0.005% alizarin red [Alfa Aesar, 42746, Ward Hill, MA, USA], 1% KOH in PBS) for 4 h. They were then cleared overnight with 1:1 glycerol:1% KOH and stored in 70% glycerol in PBS.

### 2.10. Microscopes and Imaging

Alcian blue preps were imaged using a Meiji EMZ stereo microscope with LED epi- and trans-illumination and a Canon Vixia HF S21 camera. Images to document gross morphology and *mylz2:GFP* larvae were captured using a Leica MZ-FLIII fluorescence stereo microscope and a Martin HD video camera (Martin Microscope Company; Easley, SC, USA). All images for immunofluorescence were captured using a Motic BA410T Elite compound microscope equipped with epifluorescence and a Martin HD video camera.

### 2.11. Statistical Analyses

For comparisons between two samples (heart rate, hatching rate, and caspase-3 fluorescence intensity experiments), the Student’s two-tailed t-test was used. Analyses were performed using JMP Pro 14 or Microsoft Excel, and *p* < 0.05 was used as the threshold to indicate statistical significance. For comparisons between three samples (touch response assay), Chi-square tests were performed using online software (https://www.socialscistatistics.com/tests/chisquare2/default2.aspx, accessed on 2 October 2020), and *p* < 0.05 was used as the threshold to indicate statistical significance.

## 3. Results

### 3.1. APAP-Treated Larvae Exhibit Broad Morphological and Physiological Defects

**Figure 1:**
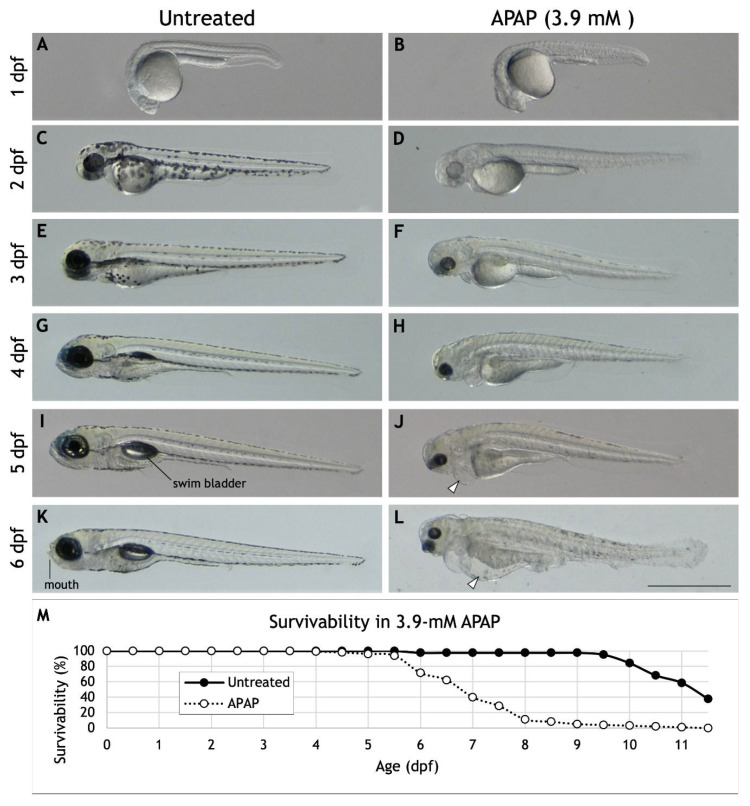
Morphology and survivability of APAP-treated larvae. (**A**–**L**) Lateral views of a single, representative larva either untreated (**A**,**C**,**E**,**G**,**I**,**K**) or continuously treated with 3.9-mM APAP from 2 hpf (**B**,**D**,**F**,**H**,**J**,**L**). Previously reported abnormalities visible here include reduced pigmentation, deformed tail and fin, failure to inflate swim bladder (compare (**I**) and (**J**)), peritoneal edema (arrowhead in (**J**)), and pericardial edema (arrowhead in (**L**)). Note the absence of a visible jaw or mouth in the APAP-treated larva (compare (**K**) and (**L**)). Many other larvae (not shown) also exhibit reduced body length and severe spinal curvature as reported elsewhere. Scale bar (in (**L**)) for (**A**–**L**), 1 mm. (**M**) Survivability of untreated larvae (*n* = 88) and larvae continuously treated with 3.9-mM APAP from 2 hpf (*n* = 98). The longest-lived treated larva survived until 11.5 dpf.

Several previous studies in zebrafish have reported a broad array of morphological and physiological defects resulting from APAP exposure over a range of concentrations spanning more than five orders of magnitude [8,9,10,11,12,13,14,15] (Table S1). As part of our own screening project, we settled on 3.9-mM APAP as a suitable treatment since it was our highest concentration tested that did not cause any significant death by 5 dpf. 3.9-mM APAP treatment also recapitulated many of the defects previously reported such as spinal curvature, reduced pigmentation, deformed tail and fin, failure to inflate swim bladder, peritoneal edema, and pericardial edema (Figure 1A–L). Despite these morphological defects, conflicting data exists on the effects of APAP on hatching behavior, with some reporting delayed hatching [13,15] and others reporting normal hatching behavior [9,10]. Using 3.9-mM APAP, we found no significant difference in hatching behavior (Figure S1) when meticulously cared for to ensure high survivability. We also probed for sensorimotor defects using a touch response assay [27] and observed that despite normal hatching behavior, APAP-treated embryos were substantially less responsive to head or tail touches (Figure S2). And despite virtually no death within the first several days, APAP-treated embryos showed reduced heart rates from 1–3 dpf (Figure S3). Survivability dropped off steadily beginning at 6 dpf, with our longest-lived larva surviving through 11.5 dpf. Together, these results suggest that high APAP dosage disrupts the development of multiple systems, leading to widespread developmental abnormalities.

Most striking to us, however, was the reduction in head size and lack of any discernable jaw or mouth structures in APAP-treated larvae (Figure 1K,L). Therefore, to test the extent of these craniofacial abnormalities, we analyzed craniofacial cartilage development.

### 3.2. APAP Disrupts the Development of Pharyngeal Arch-Derived Cartilages

**Figure 2:**
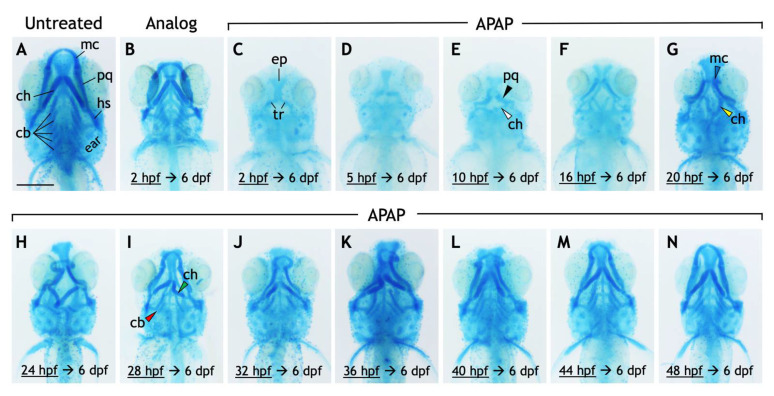
Exposure to acetaminophen, but not a non-hepatoxic analog, disrupts the development and morphology of craniofacial cartilages. (**A**–**N**) Ventral views of 6-dpf larvae stained with alcian blue. Compared to untreated larvae (**A**), those treated continuously with 3-acetamidophenol (3.9 mM), a non-hepatotoxic acetaminophen analog (**B**), have a reduced body size but with all craniofacial cartilages intact. (**C**–**N**) APAP treatment (3.9 mM) was initiated at the timepoints indicated then continuously through 6 dpf. The effects on the cartilages of the viscerocranium (labeled in (**A**)), in both their presence and morphology, are generally less severe as the initiation of treatment is delayed (**C**–**N**). In a larva treated with APAP from 2 hpf (**C**), all cartilages of the viscerocranium are missing, making visible cartilages of the neurocranium, most prominently the ethmoid plate (ep) and the bilateral trabeculae (tr). These cartilages were clearly visible in all larvae in all treatment groups. Incrementally delaying treatment results in the restoration of cartilages derived from the first and second pharyngeal arches (e.g., Meckel’s cartilage) earliest, followed by cartilages derived from the third through seventh pharyngeal arches (ceratobranchial cartilages). With treatment delayed to 10 hpf, the palatoquadrate (pq) and ventral ceratohyal (ch) cartilages are more frequently present (black and white arrowheads in E, respectively). When treatment is delayed to at least 20 hpf, the ventral ceratohyal cartilages tend to be oriented posteriorly rather than anteriorly (compare yellow arrowhead in (**G**) with morphology in (**A**)), and Meckel’s cartilage (mc) becomes more frequent (blue arrowhead in (**G**)) though misshapen. The ventral ceratohyal cartilages also begin to be oriented anteriorly (green arrowhead in (**I**)). Treatment at 28 hpf restores a reduced number of ceratobranchial cartilages in most larvae (red arrowhead in (**I**)), though they can appear as early as with a 24 hpf treatment. One representative larva is shown from each treatment group, though the effects vary within each treatment group. Scale bar (in (**A**)) for (**A**–**N**), 250 µm.

To examine whether APAP disrupts craniofacial cartilage development, we stained 6-dpf larvae with alcian blue. Remarkably, APAP-treated larvae showed defects in pharyngeal arch-derived cartilages of the viscerocranium (Figure 2). The ethmoid plate and trabecular cartilages of the neurocranium were clearly visible in all larvae. In contrast, all five ceratobranchial cartilages, which comprise part of the future gill cartilages and are derived from pharyngeal arches three through seven, failed to form in all treated larvae. More anteriorly, the effects were variable but severe. Meckel’s cartilage and the palatoquadrate cartilages, which comprise the jaw and are derived from the first pharyngeal arch, failed to form in many APAP-treated larvae. The ventral ceratohyal and dorsal hyosymplectic cartilages, both derived from the second pharyngeal arch, were likewise missing in many larvae. Interestingly, the ventral ceratohyal cartilages, which are normally oriented anteriorly and join at the midline, were instead oriented posteriorly in many larvae. To test the specificity of these defects to APAP, we also treated larvae with 3.9-mM 3-acetamidophenol, a non-hepatotoxic analog of APAP [4]. Despite a reduction in head size, all craniofacial cartilage types were intact in all analog-treated larvae (Figure 2B). Together, these results suggest that APAP disrupts the development of craniofacial cartilages, most prominently the pharyngeal arch-derived cartilages of the viscerocranium. 

**Figure 3:**
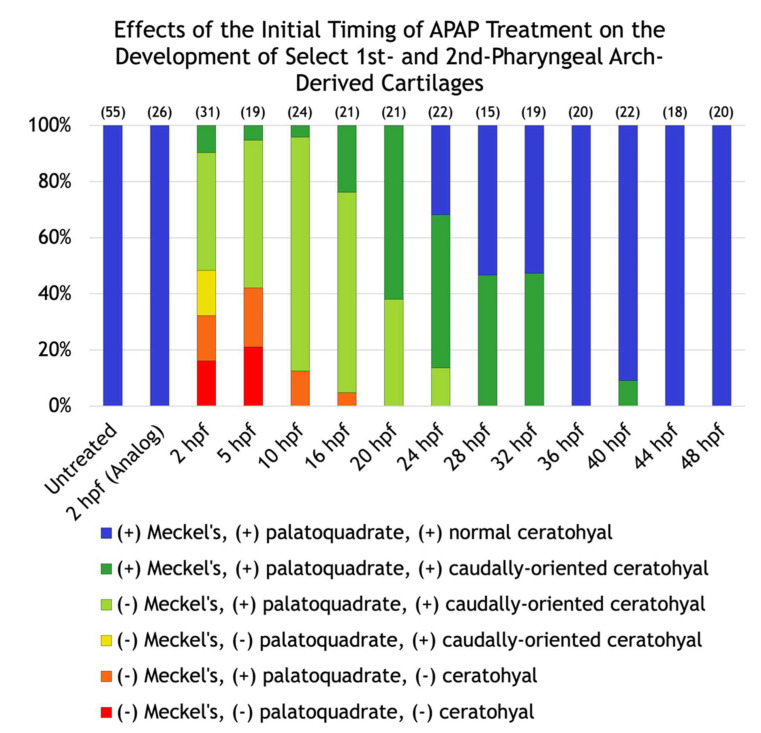
Effects of the initial timing of APAP treatment on the development of select first and second pharyngeal arch-derived cartilages. A quantification of the defects shown in Figure 2. The number of alcian blue-stained larvae analyzed for each group are in parentheses at the top of each column.

**Figure 4:**
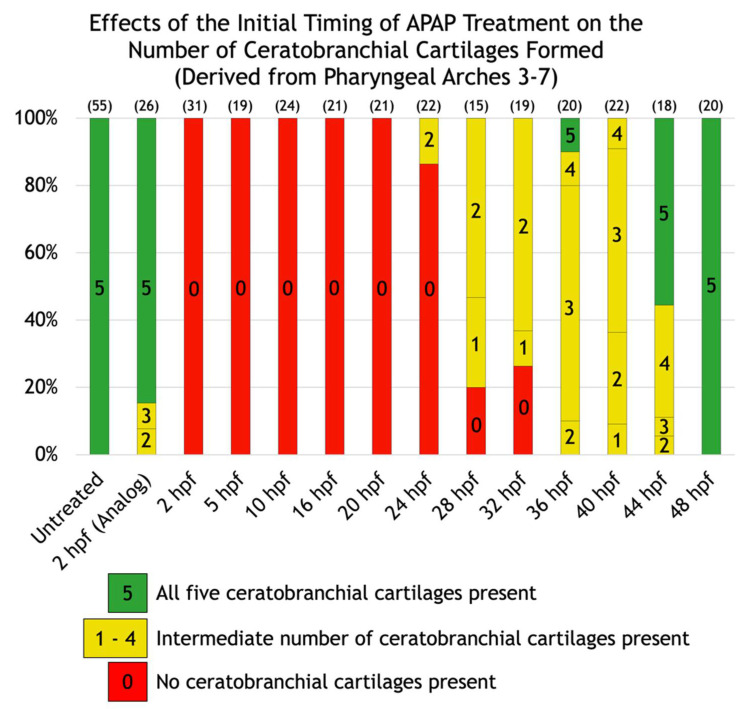
Effects of the initial timing of APAP treatment on the number of ceratobranchial cartilages formed (derived from pharyngeal arches three through seven). A quantification of the defects shown in Figure 2. The number of ceratobranchial cartilages formed are indicated within each bar. Green bars indicate all five ceratobranchial cartilages are present, yellow bars indicate an intermediate number, and red bars indicate none are present. The number of alcian blue-stained larvae analyzed for each group are in parentheses at the top of each column.

To gain insight into the nature of these variable defects, we scored the defects in each larva and placed it into one of six different categories based on whether the Meckel’s, palatoquadrate, and/or ceratohyal cartilages were present; and if present, whether the ceratohyal cartilages were oriented anteriorly (as normal) or posteriorly. In addition, to test whether different cartilages become insensitive to APAP at different timepoints, we incrementally delayed the onset of treatment (Figure 2C–N) and quantified its effects. A significant pattern emerged. As treatment was delayed, missing cartilages were generally restored in an anterior-to-posterior direction (Figure 3). Initial treatments at 5 or 10 hpf tended to restore proper orientation of the ceratohyal cartilages. With an initial treatment at 16 hpf, Meckel’s cartilage began to appear in more larvae, with full restoration in all larvae by a 28-hpf initial treatment. Thus, cartilages derived from pharyngeal arches one and two are the earliest to become insensitive to APAP treatment, but still prior to the end of chondrocyte differentiation, suggesting that APAP targets an earlier process such as pharyngeal arch development. In further support of this, ceratobranchial cartilages (derived from pharyngeal arches three through seven) were sensitive to APAP treatment for a much longer period (Figure 4). Only when treatment was delayed until 24 hpf did the first ceratobranchial cartilages appear in any larvae, with a full restoration of all five ceratobranchials occurring only when treatment was delayed until 48 hpf.

### 3.3. APAP Treatment Causes Apoptosis in the Head Region during Pharyngeal Arch Development

**Figure 5:**
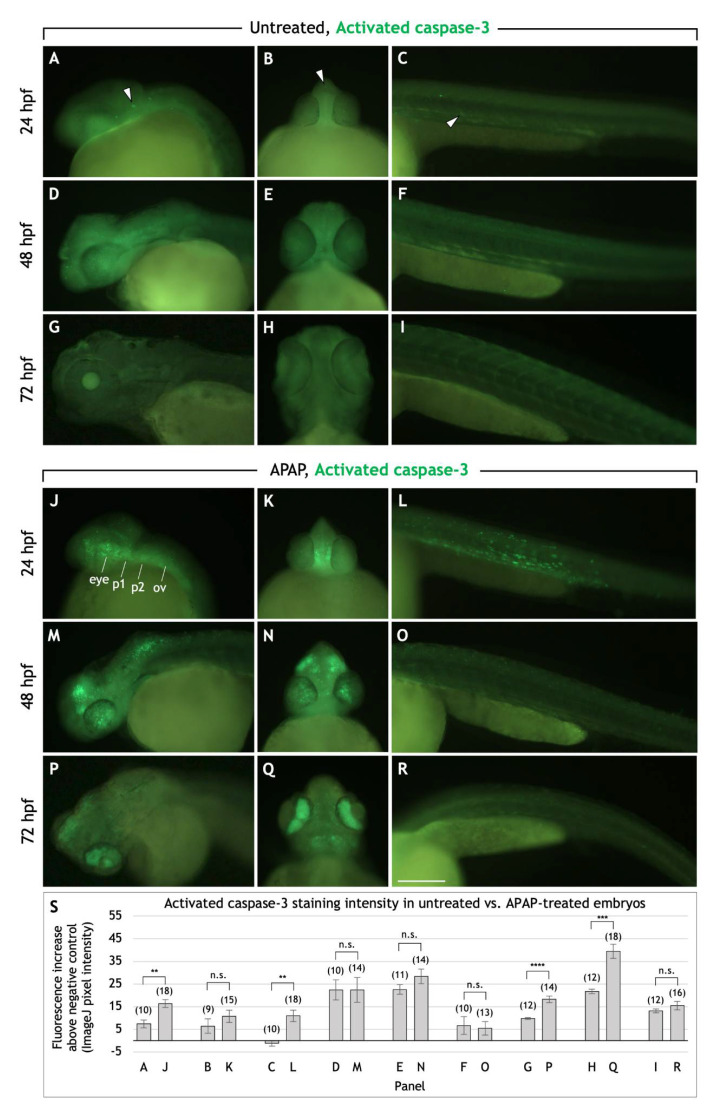
APAP treatment increases apoptosis in the head and pharyngeal arches before and during cartilage differentiation. Lateral and rostroventral views of the head and trunk regions in 24, 48, and 72 hpf embryos processed for anti-activated caspase-3 immunohistochemistry to detect apoptotic cells. In untreated embryos, apoptotic cells are sparsely distributed in the head and trunk (white arrowheads in (**A**–**C**) as examples). In APAP-treated embryos at 24 hpf (**J**–**L**), apoptosis is substantially increased between the eyes (**K**) and along the length of the head (**J**), in the regions where the mandibular and hyoid arches (1st and 2nd pharyngeal arches) are forming. Apoptotic cells are also increased throughout the trunk (**L**), but by 48 and 72 hpf, are similar to control levels (**O**,**R**). At 48 hpf, large clusters of apoptotic cells are located in the dorsal head region and throughout the eye (**M**,**N**). At 72 hpf, apoptotic cells are concentrated in the iris, with significant numbers also between the eyes and at the base of the head. Abbreviations: First pharyngeal arch, b1; second pharyngeal arch, b2; otic vesicle, ov. Scale bar (in (**R**)) for (**A**–**R**), 200 µm. (**S**): Quantification of staining intensity across samples represented in panels A-R. Lateral views of the head (**A**,**D**,**G**,**J**,**M**,**P**) were analyzed by measuring pixel intensity within the head region from the rostral-most portion through the otic vesicle. For ventral views of the head (**B**,**E**,**H**,**K**,**N**,**Q**), pixel intensity was measured within the entire head anterior to the yolk. For lateral views of the trunk (**C**,**F**,**I**,**L**,**O**,**R**), pixel intensity was measured within the trunk region dorsal to the yolk extension. For all images, negative control values were subtracted from actual values in order to report the overall fluorescence increase. The total number of embryos tested are in parentheses. ** = *t*-test at *p* < 0.01. *** = *t*-test at *p* < 0.001. **** = *t*-test at *p* < 0.0001. n.s. = not significant (*p* > 0.05). Error bars indicate the standard error of the mean.

We wondered whether the reduced body and head size of APAP-treated larvae, as well as the significantly lighter alcian blue staining (see Figure 2C) could be due to an increase in apoptosis and therefore account for the defects. For this and all following experiments, APAP treatment was initiated by 2 hpf. Staining of live 24-hpf embryos with acridine orange (AO) showed that APAP treatment significantly increased apoptosis throughout the body (Figure S4). To gain better resolution in the head region and at different timepoints throughout cartilage development, we labeled apoptotic cells using an anti-activated caspase-3 antibody (Figure 5) [31]. At 24 hpf, consistent with AO staining, apoptotic cells were increased in number, particularly in the head region. Clusters of apoptotic cells were prevalent laterally in the region of the developing pharyngeal arches (Figure 5J), and the overall increase in fluorescence intensity in this region and in the trunk was statistically significant (Figure 5S). By 48 hpf, trunk apoptosis returned to normal levels but was found in the anterior neurocranium and eyes, and not in any pattern that would suggest a targeting of chondrogenesis (Figure 5M–O). By 72 hpf, apoptotic cells were mostly concentrated in the iris but also sparsely throughout the head (Figure 5P,Q). Both lateral and ventral views of the head showed a statistically significant increase in fluorescence intensity (Figure 5S). Overall, these patterns of apoptosis suggest that APAP may interfere with viscerocranial cartilage development by inducing cell death in the pharyngeal arches, not in developing cartilages themselves.

### 3.4. Craniofacial Cartilage Defects in APAP-Treated Larvae Are Likely Not a Consequence of Defective Neural Crest or Cartilage Differentiation

Given the absence of viscerocranial cartilages in APAP-treated larvae (Figure 2C) and increased head apoptosis at 24 hpf (Figure 5J,K), well before cartilage differentiation, we wondered whether cartilage differentiation takes place at all. Craniofacial cartilages are derived from cranial neural crest cells (NCCs) which initially express sox9a and sox10 and migrate into the pharyngeal arches. Cartilages of the viscerocranium (e.g., Meckel’s) maintain sox9a expression throughout chondrogenesis as they are derived from the pharyngeal arches, whereas cartilages of the neurocranium (e.g., ethmoid plate, trabeculae) are derived from NCCs that migrate out of the first pharyngeal arch and maintain both sox9a and sox10 expression [18,20,32]. Any misexpression of sox9a or sox10 could account for the defects seen in APAP-treated larvae; therefore, we examined sox9a and sox10 expression.

**Figure 6:**
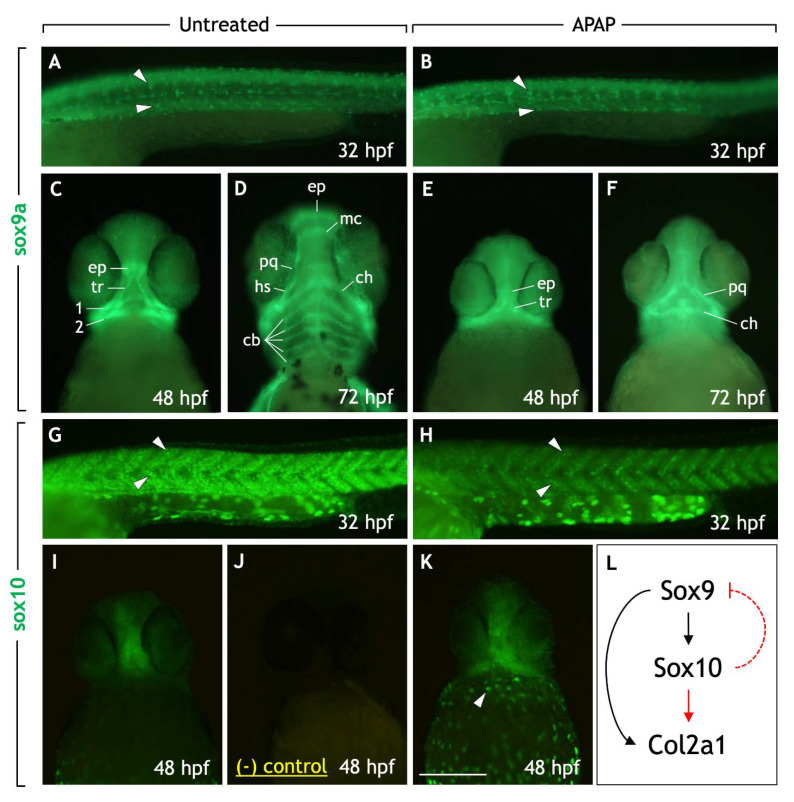
Neural crest cell markers sox9a and sox10 are expressed in trunk and cranial neural crest cells in APAP-treated embryos. (**A**–**K**) Lateral views (**A**,**B**,**G**,**H**) or ventral views (**C**–**F**,**I**–**K**) of embryos processed for sox9a (**A**–**F**) or sox10 (**G**–**K**) immunohistochemistry. sox9a-positive trunk NC cells can be seen migrating ventrally in both untreated (**A**) and APAP-treated (**B**) embryos at 32 hpf (white arrowheads). In the head at 48 hpf (**C**) and 72 hpf (**D**), sox9a labels NC-derived prechondrogenic cells. In APAP-treated embryos (**E**,**F**), sox9a expression is reduced due to missing viscerocranium/pharyngeal arch-derived cartilage types. At 32 hpf, sox10-positive trunk NC cells migrate ventrally in streams in untreated embryos ((**G**), white arrowheads). In APAP-treated embryos (**H**), sox10-positive cells are reduced in number but follow similar migration paths (white arrowheads). In the head at 48 hpf (**I**), sox10 is expressed along the midline of the neurocranium, verified by comparison with negative control embryos in which the same secondary antibody (but no primary antibody) was applied (**J**). In APAP-treated embryos, sox10 expression is similar but more robust, with enhanced expression along the yolk sac (white arrowhead). (**L**) An overview of a basic gene regulatory network in NC-derived cartilage precursor cells in chick (from Suzuki 2006). Red lines indicate interactions that have not been established in zebrafish. In early NC cells, Sox9 induces Sox10 expression, which in turn downregulates Sox9. In cartilage precursors, Sox10 expression is downregulated causing the recovery of Sox9 expression. Together, both Sox9 and Sox10 positively regulate Col2a1 expression, a marker for differentiating chondrocytes. Abbreviations: ceratobranchial cartilages, cb; ventral ceratohyal, ch; ethmoid plate, ep; dorsal hyosymplectic, dh; Meckel’s cartilage, mc; palatoquadrate, pq; trabecular, tr. Scale bar (in (**K**)) for (**A**–**K**), 200 µm.

In both untreated and APAP-treated embryos, comparable numbers of sox9a-positive NCCs could be seen migrating ventrally along the somites throughout the trunk region at 32 hpf (Figure 6A,B). In the head, sox9a was expressed in chondrocytes as the various cartilage elements arose between 48–72 hpf in untreated embryos (Figure 6C,D). In APAP-treated embryos, however, sox9a was mostly absent from the viscerocranium, while expressed normally in developing neurocranial chondrocytes (Figure 6E,F). This is consistent with the reduction in viscerocranial cartilages seen in the alcian blue preps at later stages (Figure 2). Therefore, while sox9a expression is intact in the trunk and neurocranium, the absence of sox9a in the viscerocranium could account for the downstream failure of cartilage development.

Similar to the above, sox10-positive NCCs could be seen migrating in the trunk region in both untreated and APAP-treated embryos at 32 hpf (Figure 6G,H), albeit in reduced numbers in APAP-treated embryos. In the head, sox10 expression was maintained in anterior neurocranial cartilages at 48 hpf [33]. In both untreated and APAP-treated 48-hpf embryos, sox10 was similarly expressed in the ethmoid plate and trabecular cartilages of the anterior neurocranium (Figure 6I,K). Interestingly, in APAP-treated embryos, additional sox10 expression was consistently seen in the posterior head and appeared upregulated in pigment cells migrating around the yolk sac. This is consistent with the reported upregulation of whole-embryo sox10 RNA levels in APAP-treated embryos [8] and suggests the possibility that downstream genes involved in chondrogenesis could be misregulated.

**Figure 7:**
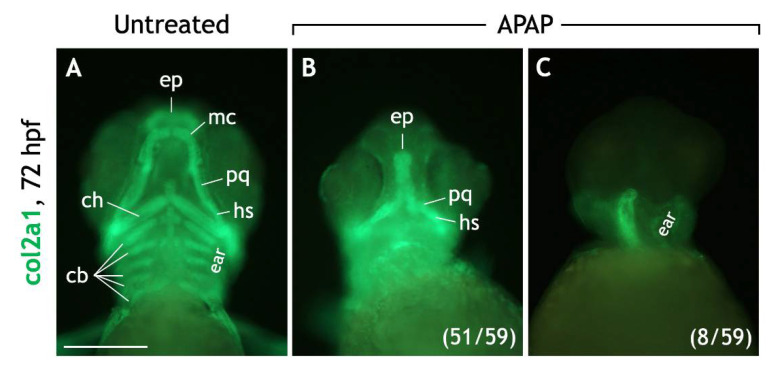
Most APAP-treated embryos express collagen type II, a marker for differentiated cartilage, in the craniofacial region. (**A**–**C**) Ventral views of 72-hpf embryos processed for col2a1 (collagen type II) IHC. Untreated embryos (**A**) express col2a1 in all craniofacial cartilages. In most APAP-treated embryos (**B**), col2a1 expression is equally robust but reflects the absence of numerous cartilage types. Diffuse col2a1 expression is consistently observed around the developing ears and at the base of the head. In other APAP-treated embryos (**C**), col2a1 expression is completely absent in the anterior head region. The numbers of col2a1-labeled, APAP-treated embryos are labeled in parentheses. Abbreviations: ceratobranchial cartilages, cb; ventral ceratohyal, ch; ethmoid plate, ep; dorsal hyosymplectic, dh; Meckel’s cartilage, mc; palatoquadrate, pq. Scale bar (in (**A**)) for (**A**–**C**), 200 µm.

To further test chondrocyte differentiation, we examined collagen type II (col2a1) expression. col2a1 is expressed by sox9a-positive NCCs as they condense to form cartilage [34] and is directly regulated by sox9a/b [35]. Sox10 is also required for Col2a1 expression in chick [36], but this has not been established in zebrafish. In untreated embryos at 72 hpf, col2a1 was expressed by all craniofacial cartilages (Figure 7A). However, in APAP-treated embryos, col2a1 was variably expressed. In most embryos (51/59), col2a1 was expressed by neurocranial cartilages and often the palatoquadrate/dorsal hyosymplectic cartilages of the viscerocranium (Figure 7B). Interestingly, in some embryos (8/59), col2a1 was completely absent from the anterior head (Figure 7C). Together, these results suggest that although many NCCs and developing cartilages express sox9a/sox10, there is likely a breakdown of the differentiation program in some embryos resulting in a loss of col2a1 expression. 

### 3.5. Craniofacial Muscle Development Is Disrupted in APAP-Treated Larvae

**Figure 8:**
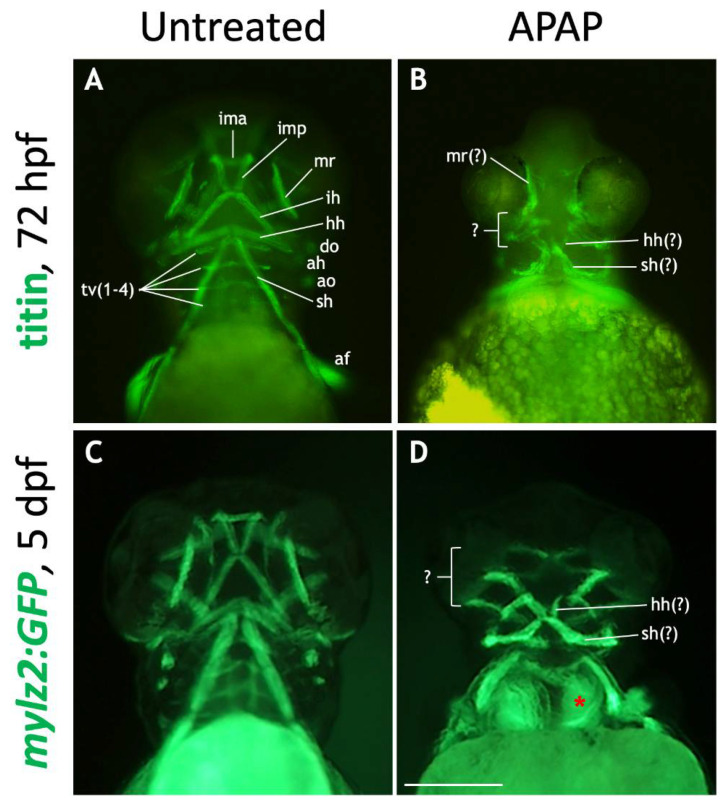
Many craniofacial muscles are absent or disorganized in APAP-treated larvae. (**A**,**B**): Ventral views of 72-hpf embryos processed for titin immunohistochemistry to label muscles. In an untreated embryo (**A**), ventral muscle types are in focus and labeled according to type. In an APAP-treated embryo (**B**), developing muscles are present but disorganized. (**C**,**D**): Ventral views of live-imaged, 5-dpf mylz2:GFP larvae which express GFP in skeletal muscles. Compared to an untreated larva (**C**), an APAP-treated larva (**D**) has a reduced number of misarranged craniofacial muscles. Due to missing musculoskeletal elements from branchial arches three though seven, the underlying anterior somites are visible (red asterisk in (**D**)). The defects in both titin-stained and mylz2:GFP larvae are variable, but the most common arrangements are shown here. Abbreviations (after Schilling and Kimmel 1997): aboral fin, af; adductor hyoideus, ah; adductor opercula, ao; dilator opercula, do; hyohyoideus, hh; interhyoideus, ih; intermandibularis anterior, ima; intermandibularis posterior, imp; medial rectus, mr; sternohyoideus, sh; transversus ventralis, tv. Scale bar (in (**D**)) for (**A**–**D**), 200 µm.

Given the close association between developing chondroblasts and myoblasts and their likely interdependency [37], we probed for muscular defects in APAP-treated embryos. As expected, APAP treatment caused severe defects. First, we immunostained 72-hpf embryos for titin (Figure 8A,B), which labels skeletal muscle [38]. In untreated embryos, titin labeled all of the major cranial muscles (of the neurocranium) and pharyngeal muscles (of the viscerocranium) (Figure 8A). However, in APAP-treated embryos, most muscle types were missing, and those remaining were severely disorganized as to make their exact identification difficult (Figure 8B). This is in contrast to results seen with cartilage staining (Figure 2), where individual cartilage types could be easily identified. To visualize developing muscles at a later timepoint where whole-mount immunostaining is unreliable, and to test whether muscle development might be delayed, we utilized the *mylz2:GFP* transgenic line [23]. At 5 dpf, GFP was expressed in skeletal muscle, including all craniofacial muscles (Figure 8C). However, in APAP-treated larvae, muscles were disorganized, and many were missing, notably those associated with the ceratobranchial cartilages and the lower jaw (Figure 8D). This is consistent with the cartilage defects (Figure 2) and suggests that muscle development is hindered by the absence of various craniofacial cartilages in APAP-treated larvae.

### 3.6. Repeated Administration of APAP to Pregnant Mice Does Not Recapitulate Craniofacial Defects

Given the similarities in APAP metabolism between zebrafish and mammals, we wondered whether APAP could cause craniofacial defects in mice. Therefore, we administered APAP to timed-pregnant female mice during timepoints corresponding to neural crest migration (embryonic day [E]7.25–10.75) or cartilage condensation (E10.25–13.75) [39]. APAP doses (or saline control doses) were administered via oral gavage three times daily. In our preliminary trials, administration of 250 mg/kg APAP three times daily between E10.25–13.75 caused miscarriage in confirmed pregnant females (*n* = 4). This was not surprising due to the known placental damage that occurs with even a single 250 mg/kg APAP dose [40]. Therefore, we lowered the dose to 200 mg/kg three times daily, which roughly corresponds to a human equivalent dose of 2800 mg/day, slightly under the maximum recommended daily dose of 3000 mg/kg. The resulting pups were collected at postnatal stage (P)0 and processed for dual bone/cartilage skeletal staining [30].

**Figure 9:**
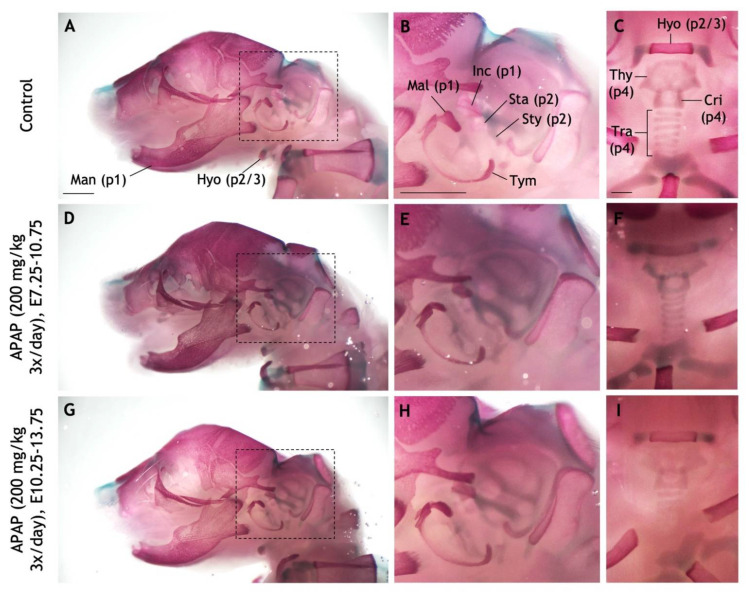
Repeated administration of high APAP dosage to pregnant mice does not recapitulate craniofacial defects. (**A**–**I**) Pregnant dams were administered via oral gavage three times daily: Saline between E10.25–13.75 (**A**–**C**), 200 mg/kg APAP between E7.25–10.75 (**D**–**F**), 200 mg/kg APAP between E10.25–13.75 (**G**–**I**). The resulting pups were collected at P0 and stained with alcian blue (cartilage) and alizarin red (bone). (**A**–**C**) A control pup with pertinent bones and cartilages labeled. Pharyngeal arch-derived structures are indicated with parentheses (e.g., “p1” = derived from the first pharyngeal arch). (**D**–**F**) In a pup whose mother was treated with APAP between E7.25–10.75, all labeled structures develop normally. (**G**–**I**) No defects seen in a pup whose mother was treated with APAP between E10.25–13.75. Dashed boxes in (**A**,**D**,**G**) are the fields of view for panels (**B**,**E**,**H**), respectively. Abbreviations: cricoid cartilage, Cri; hyoid, Hyo; incus, Inc; malleus, Mal; mandible, Man; stapes, Sta; styoid process of the temporal bone, Sty; thyroid cartilage, Thy; tracheal cartilages, Tra; tympanic, Tym. Scale bars in (**A**–**C**), 1 mm.

In saline-gavaged control pups, all pharyngeal arch-derived cartilages and bones were present and easily visualized (Figure 9A–C). These included the mandible, malleus, and incus (pharyngeal arch one); the stapes, and styoid process of the temporal bone (pharyngeal arch two); the hyoid bone (pharyngeal arches two and three); the thyroid cartilage, cricoid cartilage, and tracheal cartilages (pharyngeal arch four). In pups gavaged with 200 mg/kg APAP three times daily between E7.25–10.75 (Figure 9D–F) and E10.25–13.75 (Figure 9G–I), all of the above bones/cartilages were present and of normal morphology. These results suggest that at this biologically relevant dose, APAP does not cause craniofacial defects in mice, and that the maximum dosage appears to be limited by its effects on the placenta.

## 4. Discussion

In this study, we demonstrate that high APAP exposure causes craniofacial defects in zebrafish, and importantly, that pharyngeal arch-derived cartilages and muscles are primarily affected. Patterns of apoptosis in the head suggest that APAP may target early pharyngeal arch development. Though many migrating neural crest cells (NCCs) and their cranial chondrocyte derivatives continue to express sox9a/sox10 as expected, some APAP-treated embryos exhibit a breakdown in cartilage differentiation as exemplified by their failure to express col2a1 in the head. This analysis provides a basis for uncovering the molecular mechanisms of APAP interference with craniofacial development.

### 4.1. APAP Causes Broad Morphological and Physiological Defects

Various studies in zebrafish have reported broad morphological and physiological defects caused by APAP exposure (Table S1), some of which we verify here (Figure 1 and Figures S1–S3). These studies have used a wide range of APAP concentrations from which an interesting trend emerges. Morphological defects such as spinal curvature, reduced pigmentation, and edema have been reported with environmental APAP concentrations as low as 4 × 10^−3^ mM, 4 × 10^−3^ mM, and 3.2 × 10^−3^ mM, respectively [9,12]. Physiological defects in touch response, auditory and photo responses, and equilibrium have been reported with as low as 6.5 × 10^−2^ mM, 6.6 × 10^−2^ mM, and 3.2 × 10^−3^ mM, respectively [9,13,15]. Though the absolute minimum concentrations needed to cause these defects are not known, it is clear that substantially higher APAP exposure is required to cause craniofacial defects. We report here that 3.9-mM APAP causes the obliteration of or deformities in viscerocranial cartilages. Recently, Cedron et al. (2020) reported a similar result at 3.5 mM; but interestingly, 2.5-mM APAP only produced mild but statistically significant differences in the lengths of viscerocranial cartilages and a change in the angle of the ceratohyal cartilages. This could reasonably be a consequence of reduced overall cranial length [8] as well as body length and mass [15]. Regardless, it appears that craniofacial development in zebrafish is less sensitive to APAP than other processes that have been investigated.

Zebrafish are an excellent model system for developmental toxicology, and they produce many well-known drug-metabolizing enzymes [5]. However, since compounds are typically delivered via external media, it is often unclear how much is actually absorbed into tissues. In a study on absorption of select drugs into zebrafish larval tissues, absorption was found to be related to the cLogP value of the compound (a measure of hydrophilicity), with a cLogP of <3.8 displaying low tissue penetration [41]. Based on this, APAP (cLogP = 0.49) would be predicted to have low tissue penetration, possibly less than 5% of media concentration after 1 h of exposure. However, this has not been specifically tested.

The molecular mechanism of APAP interference with development is also an open question. Like humans and mice, the zebrafish liver converts APAP to NAPQI, a hepatotoxic metabolite [7]. This leads to the question of whether APAP-induced developmental defects might be secondary to hepatotoxicity. Some key observations argue against this. Zebrafish liver progenitors are detectable as early as 22 hpf [42] but the liver is not significantly vascularized until 72 hpf [43], which is critical for its function. Since cartilage differentiation takes place before 72 hpf and APAP-induced craniofacial cartilage defects are detectable by 48 hpf (Figure 6E), it is unlikely that they are a consequence of hepatotoxicity. Furthermore, *cyp3a65*, which encodes the enzyme likely responsible for phase I metabolism of APAP [7], is not expressed until 3 dpf [44], strongly suggesting that NAPQI is not responsible for the defects. Instead, APAP-induced developmental defects are likely due to another APAP metabolite, or perhaps even unmetabolized APAP.

### 4.2. Effects of APAP Exposure on Craniofacial Development

Our data demonstrate that continuous APAP exposure eliminates or severely disrupts pharyngeal arch-derived cartilages of the viscerocranium (Figure 2). As exposure is delayed, cartilage morphology is restored in the order of pharyngeal arch number, such that cartilages derived from pharyngeal arches one and two are restored first (Figure 3), followed by arches three through seven (Figure 4). Even in the most severely affected specimens, neurocranial cartilages were intact (Figure 2C), suggesting that APAP specifically targets pharyngeal arch development rather than generally disrupting cartilage differentiation or condensation. These defects were specific to APAP (4-acetamidophenol), as exposure to an analog (3-acetamidophenol) reduced head size but did not obliterate any cartilages (Figure 2B). If the analog were indeed non-hepatotoxic, as reported in mice [4], this could suggest that the cartilage defects caused by APAP are secondary to hepatotoxicity. However, the potential hepatotoxicity of 3-acetamidophenol has not been tested in zebrafish, and it has been demonstrated to be hepatotoxic in rats and humans [45]. Therefore, APAP most likely disrupts craniofacial development independent of liver toxicity.

APAP is one of only a few compounds examined that significantly disrupts the formation of viscerocranial cartilages in zebrafish, at least at the concentrations tested. Most compounds affecting craniofacial cartilage development result in a reduction in cartilage sizes and/or orientation, such as ethanol [46], dioxin [47], valproic acid [48], bisphenol A [49], bis-GMA [50], 6-OH-BDE-47 [51], perchlorate [52], atrazine [53], and several triazole and dithiocarbamate fungicides [54]. However, craniofacial cartilages are often missing in embryos treated with paclobutrazol [55], di-butyl phthalate [56], estradiol [57], and offspring of female fish exposed to topiramate [58], with the viscerocranial cartilages primarily affected. The potential connection between APAP and these compounds or their mechanism of action is not clear, but di-butyl phthalate and estradiol are known endocrine disruptors, and APAP is suspected to have endocrine disrupting properties [59].

In addition to cartilage defects, we found that APAP disrupted craniofacial muscle development (Figure 8). Myoblasts are derived from paraxial cranial mesoderm whereas the surrounding connective tissue and cartilage is derived from NCCs. During the early stages of muscle morphogenesis, myoblasts are dependent on NCC-derived cues [60]. Since APAP appears to specifically target pharyngeal arch-derived elements, muscular defects are to be expected due to the loss of signals due to absent chondrocytes.

### 4.3. Cellular Mechanisms Underlying APAP-Induced Craniofacial Defects

Recently, Cedron et al. (2020) reported a modest increase in apoptosis in APAP-treated, 48-hpf embryos. Our data confirm this observation (Figure 5O), but the overall patterns of apoptosis between 48–72 hpf (Figure 5M–R) do not appear sufficient to account for the craniofacial cartilage defects. In the head between 48–72 hpf, apoptosis is prevalent in the eyes but not in other locations specific to developing chondrocytes (Figure 5N,Q). Instead, we show that 24-hpf embryos undergo substantial amounts of apoptosis throughout the body and particularly throughout the head and regions where the pharyngeal arches are developing (Figure 5J–L,S). This is consistent with our observation that the pharyngeal arch-derived cartilages of the viscerocranium are most affected in APAP-treated larvae and suggests that defects in pharyngeal arch development may underlie the craniofacial defects. This possibility should be explored further in future work.

Since craniofacial cartilages are NCC-derived, any abnormalities in neural crest cell migration or gene expression could account for the craniofacial defects seen. Using RT-qPCR, Cedron et al. (2020) recently described enhanced *sox10* and decreased *sox9b* whole-embryo mRNA levels in 24-hpf zebrafish embryos treated with 4.9-mM APAP. This change in NCC marker expression could explain the downstream defects in cartilage development. However, since both genes are broadly expressed, the ability to use this data to account for head-specific defects is limited. Our whole mount expression data for sox9a and sox10 (Figure 6) indicate that in APAP-treated embryos, many NCCs migrated along their expected paths in the trunk and many cranial NCCs organized to form cartilages in the head. Although Cedron et al. (2020) examined *sox9b* whereas we examined sox9a, both genes have very similar expression patterns [61] and it is likely that the reported decrease in *sox9b* expression levels is due to the multiple missing cartilage types. In addition, since *sox10* levels are higher in APAP-treated embryos [8] and *sox10* is a known regulator of both *sox9* and *col2a1* (Figure 6L), it is feasible that NCC gene expression could be altered considerably. Our findings that col2a1 expression is obliterated in the anterior head of some APAP-treated embryos (Figure 7C) add weight to this suspicion, and future work should further explore this possibility.

### 4.4. APAP Does Not Cause Craniofacial Defects in Mice

Our data show that in pregnant mice, oral dosage of APAP does not recapitulate the craniofacial defects seen in zebrafish (Figure 9). However, the maximum dosage that can be administered is limited by the effects of APAP on the maternal liver and on placental development. APAP can freely cross the placenta, and even a single 250 mg/kg dose alters the functional area of the placenta and reduces maternal levels of progesterone [40]. The outcome of repeated APAP exposure on the placenta has not been investigated in detail, but in our own trials, none of our pregnant mice dosed with 250 mg/kg APAP three times daily between E10.25–13.5 produced any pups. Also, for these experiments, we opted for a realistic human-equivalent dosage regimen consisting of 200 mg/kg three times daily. Since the half-life of APAP is likely <1–4 h in mice [62], this does not constitute continuous exposure as in our zebrafish experiments. Therefore, it is difficult to make direct comparisons between the effects of APAP in zebrafish and the results in the mouse model. Interestingly though, a single 300 mg/kg APAP dose has been reported to increase the incidence of cleft palate by 80% and fetal resorption by 71% in pregnant mice treated with phenytoin, an anti-seizure drug [63]. While APAP alone does not cause cleft palates, it likely potentiates these defects through depletion of hepatic glutathione, suggesting that APAP could affect embryonic development if used in combination with other drugs. Future studies should explore this possibility.

## Data Availability

The data presented in this study are available at figshare via the following DOIs: https://doi.org/10.6084/m9.figshare.16726369, https://doi.org/10.6084/m9.figshare.16726387, https://doi.org/10.6084/m9.figshare.16726408, https://doi.org/10.6084/m9.figshare.16726432, https://doi.org/10.6084/m9.figshare.16726447, https://doi.org/10.6084/m9.figshare.16726450, https://doi.org/10.6084/m9.figshare.16726459, https://doi.org/10.6084/m9.figshare.16726468, https://doi.org/10.6084/m9.figshare.16726573, https://doi.org/10.6084/m9.figshare.16727233, https://doi.org/10.6084/m9.figshare.16727281, https://doi.org/10.6084/m9.figshare.16727329, https://doi.org/10.6084/m9.figshare.16727353, https://doi.org/10.6084/m9.figshare.16727362, and https://doi.org/10.6084/m9.figshare.16727365.

## Supplementary Materials

The following supporting information can be downloaded at: https://www.mdpi.com/article/10.3390/jdb10030030/s1, Table S1: Morphological and physiological defects previously described in zebrafish embryos/larvae exposed to acetaminophen; Figure S1: APAP treatment does not delay hatching; Figure S2: APAP-treated embryos exhibit defective touch responses; Figure S3: APAP treatment results in lower heart rates; Figure S4: APAP-treated embryos have a widespread increase in apoptosis.
