# Supplementary material for: Acetaminophen Disrupts the Development of Pharyngeal Arch-Derived Cartilage and Muscle in Zebrafish
Source: J Dev Biol. 2022 Jul 14;10(3):30. doi: 10.3390/jdb10030030 (PMC9326545; doi:10.3390/jdb10030030)
Supplement: Supplementary file 1 [file jdb-10-00030-s001.zip › jdb-1766906-supplementary.pdf]

Supplementary Material

Table S1. Morphological and physiological defects previously described in zebrafish embryos/larvae exposed to acetaminophen, excluding studies describing effects on liver and kidney. Blank cells indicate *untested* parameters or those not explicitly reported.

	Cedron et al 2020	Nogueira et al 2019	Xia et al 2017	Reuter et al 2016	Galus et al 2013	Zhang & Gong 2013	He et al 2013	Xu et al 2011	David & Pancharatna 2009
APAP concentration(s) tested (mM)	2.5 - 9.6	$3.2x10^{-5}$ - 0.020	0.032 - 3.25	$3.2x10^{-3}$ - 3.25	0.003 - 0.066	0.016 - 0.16	$1x10^{-4}$ - 10	0.025 - 5	0.0066 - 0.66
Reduced survivability	Yes	NO (at 96 hpf)			Yes	Yes	Yes		
Reduced body length & body mass									Yes
Reduced CNS & spinal MN axon length						Yes			
Spinal curvature	Yes	Yes		Yes	Yes				
Deformed tail & fin									Yes
Reduced pigmentation	Yes	Yes		Yes					Yes
Failure to inflate swim bladder				Yes					
Undetached tail at 24/48 hpf						Yes			
Delayed yolk sac absorption							Yes		
Abnormal somite numbers at 24/48 hpf						Yes			
Edema (peritoneal and/or pericardial)	Yes			Yes	Yes			Yes	
Pericardial blood accumulation	Yes								
Abnormal heart looping & reduced heart valve formation								Yes	
Reduced heart rate								Yes	
Reduced brain & eye structures				Yes					
Craniofacial cartilage defects	Yes								
Delayed hatching		NO	NO			Yes			Yes
Reduced touch response						Yes			
Abnormal spontaneous movement		NO							
Hyperactivity				NO					
Reduced auditory and photo responses									Yes
Defects in equilibrium (swimming behavior)		Yes	NO						Yes

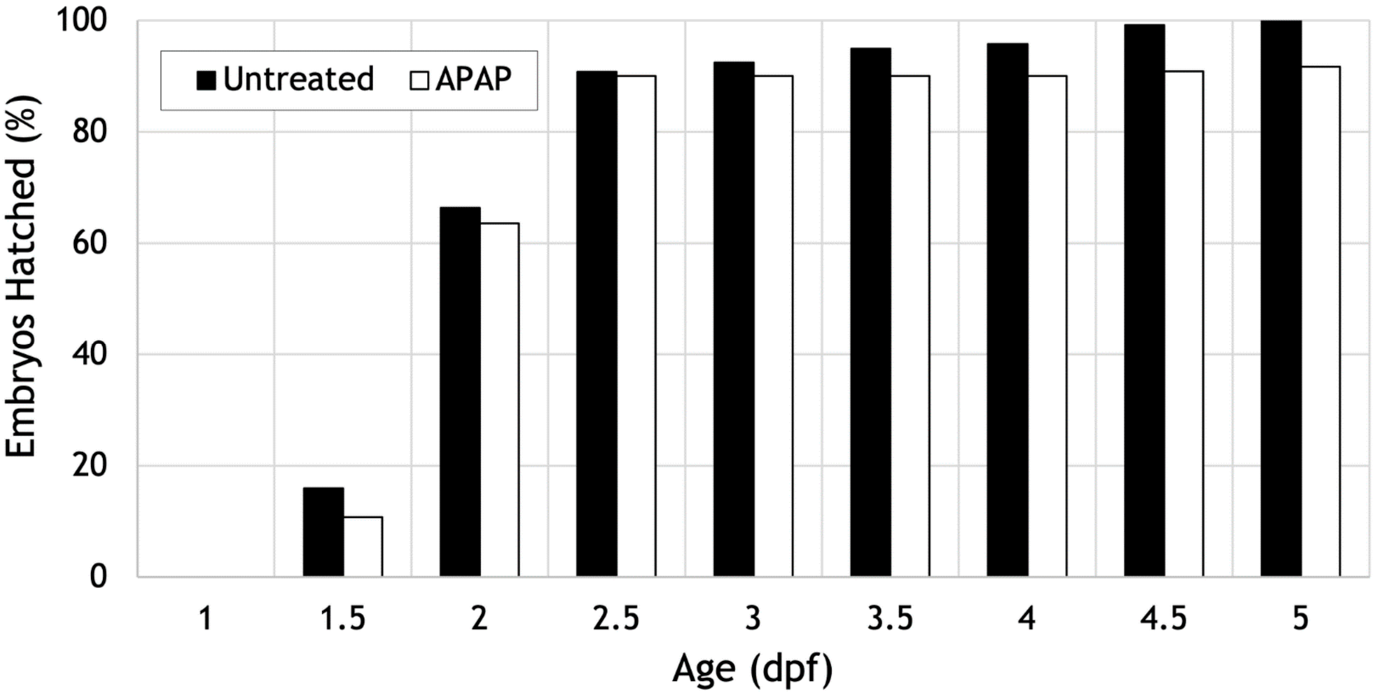

Figure S1. APAP treatment does not delay hatching. Untreated control embryos (n=119) and embryos continuously treated with 3.9-mM APAP (n=121) from 2 hpf onward were observed every 12 hours from 1 – 5 dpf. The earliest hatching occurred at 1.5 dpf, and all untreated embryos were hatched by 5 dpf. There was no significant difference between the hatching rates of untreated and APAP-treated embryos at any time point ($p > 0.05$ at all time points).

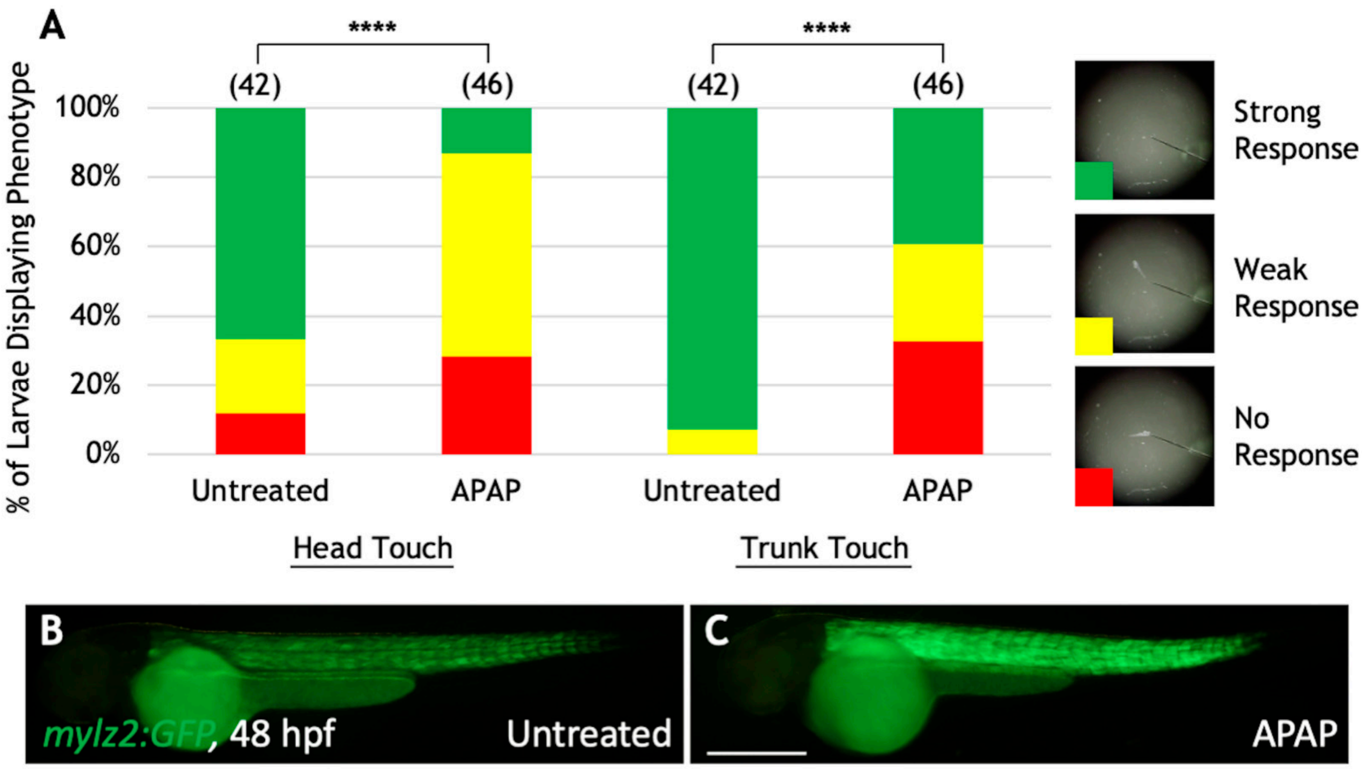

Figure S2. APAP-treated embryos exhibit defective touch responses. (A) Touch response assay. Following a single touch to either the head or trunk, 48-hpf embryo responses were scored within three categories: Strong Response (movement to outside of the field of view), Weak Response (movement remaining in the field of view), and No Response (no movement after touch). The field of view was 20 mm. APAP-treated embryos were significantly less responsive to both head and tail touches. The total number of embryos tested are in parentheses. **** = Chi-square test at $p < 0.00001$. (B-C) Lateral views of 48 hpf live-imaged *mylz2:GFP* embryos. GFP as an indicator of fast skeletal muscle is expressed in identical patterns throughout the trunk in untreated (B) and APAP-treated (C) embryos. Scale bar (in C) for B-C, 500 μm.

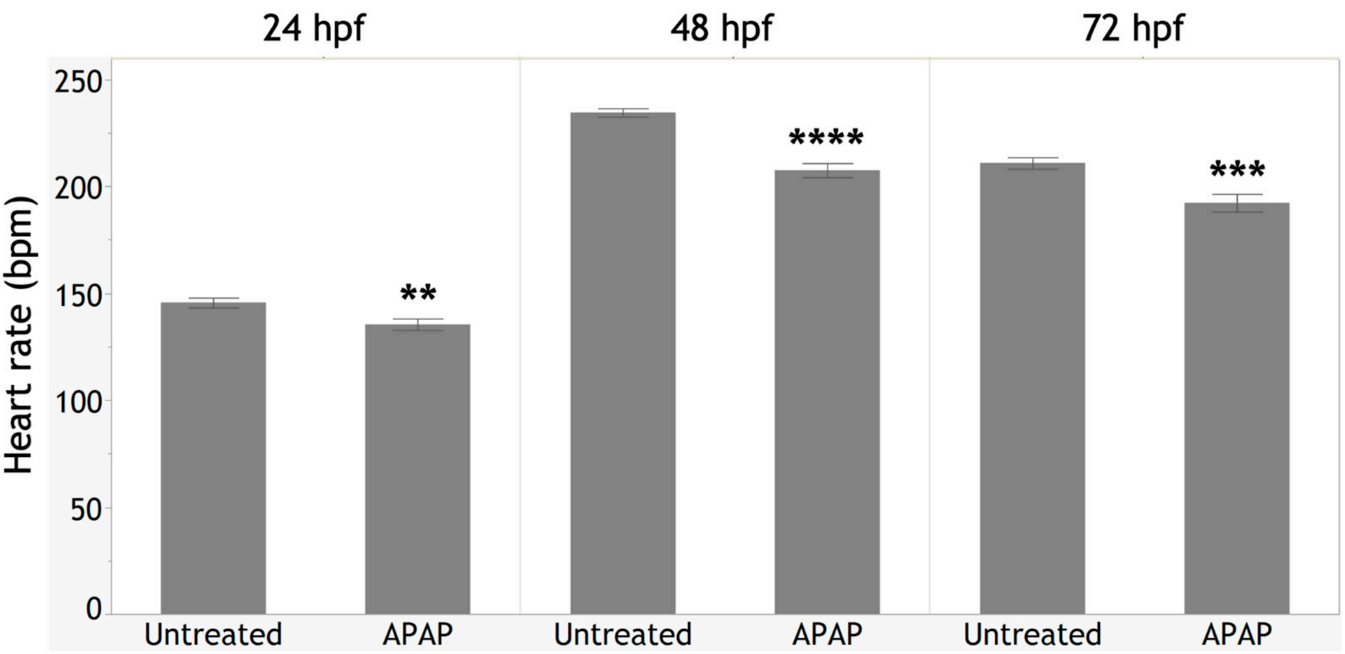

Figure S3. APAP treatment results in lower heart rates. Embryos were either continuously treated with 3.9-mM APAP (n=48) starting at 2 hpf, or untreated to serve as controls (n=48). The number of heart beats were counted for 10 seconds in each embryo, then multiplied by 6 to calculate beats per minute, represented as beats per minute ± SEM. ** = t-test at $p < 0.01$. *** = t-test at $p < 0.001$. **** = t-test at $p < 0.0001$. Error bars indicate the standard error of the mean.

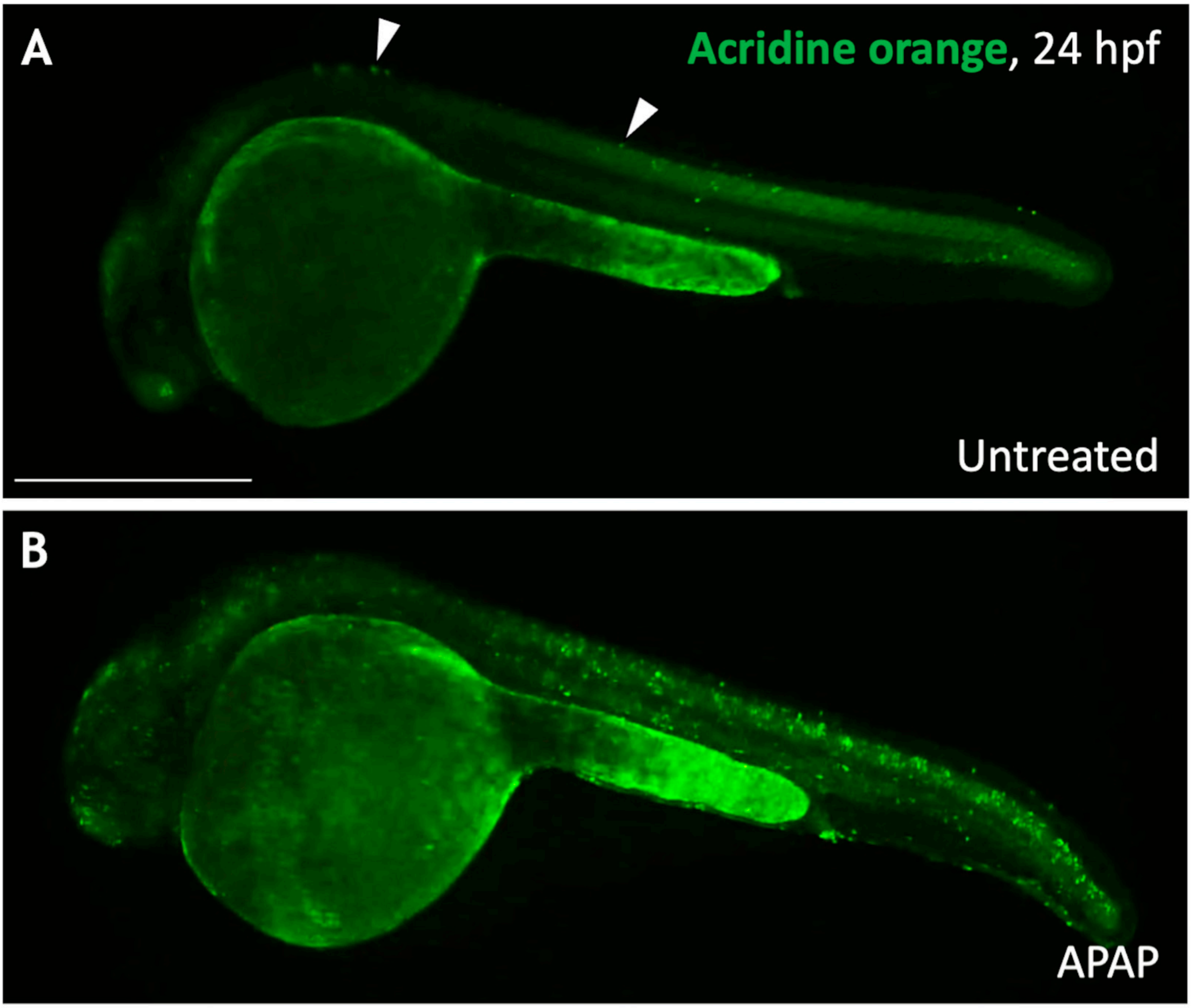

Figure S4. APAP-treated embryos have a widespread increase in apoptosis. (A-B) Lateral views of live 24-hpf embryos after incubation with acridine orange. In an untreated embryo (A), apoptotic cells (white arrowheads) are present in small numbers. In an APAP-treated embryo (B), substantially more apoptotic cells are present in both the head and tail regions. Scale bar (in A) for A-B, 500 μm.
